# Reconstitution of ATP-dependent lipid transporters: gaining insight into molecular characteristics, regulation, and mechanisms

**DOI:** 10.1042/BSR20221268

**Published:** 2023-08-09

**Authors:** Sara Abad Herrera, Thomas Günther Pomorski

**Affiliations:** 1 Department of Molecular Biochemistry, Faculty of Chemistry and Biochemistry, Ruhr University Bochum, Bochum, Germany; 2 Department of Plant and Environmental Sciences, University of Copenhagen, Frederiksberg, Denmark

**Keywords:** ABC transporter, Flipppase, Liposomes, P-type ATPase, Reconstitution

## Abstract

Lipid transporters play a crucial role in supporting essential cellular processes such as organelle assembly, vesicular trafficking, and lipid homeostasis by driving lipid transport across membranes. Cryo-electron microscopy has recently resolved the structures of several ATP-dependent lipid transporters, but functional characterization remains a major challenge. Although studies of detergent-purified proteins have advanced our understanding of these transporters, *in vitro* evidence for lipid transport is still limited to a few ATP-dependent lipid transporters. Reconstitution into model membranes, such as liposomes, is a suitable approach to study lipid transporters *in vitro* and to investigate their key molecular features. In this review, we discuss the current approaches for reconstituting ATP-driven lipid transporters into large liposomes and common techniques used to study lipid transport in proteoliposomes. We also highlight the existing knowledge on the regulatory mechanisms that modulate the activity of lipid transporters, and finally, we address the limitations of the current approaches and future perspectives in this field.

## Introduction

Cellular membranes are vital structures that maintain cell integrity and perform critical functions such as organelle compartmentalization, signaling, and membrane trafficking. These complex structures are composed of a hydrophobic matrix consisting of a double layer of lipids [1,2]. Embedded or attached to the membrane are proteins that dynamically interact with lipids to ensure proper membrane integrity and functionality [3]. While lipids can move between the two leaflets of the membrane, the rate of transbilayer lipid movement varies depending on the physical properties of the lipid and the environment. Lipids with small headgroups such as diacylglycerol, ceramide, and cholesterol can move from one leaflet to the other in artificial membranes in seconds or minutes. In contrast, lipids with polar headgroups such as phosphatidylcholine (PC) and sphingomyelin, and glycolipids with bulky hydrophilic carbohydrate moieties move more slowly, with half-times ranging from hours to days, depending on the size and charge of their headgroup [4,5]. Overall, these rates are insufficient to support vital cellular processes such as membrane assembly, control of transbilayer lipid asymmetry, and vesicular trafficking. Therefore, cellular membranes are equipped with lipid transporters that facilitate the movement of lipids across cellular membranes [6].

**Figure 1:**
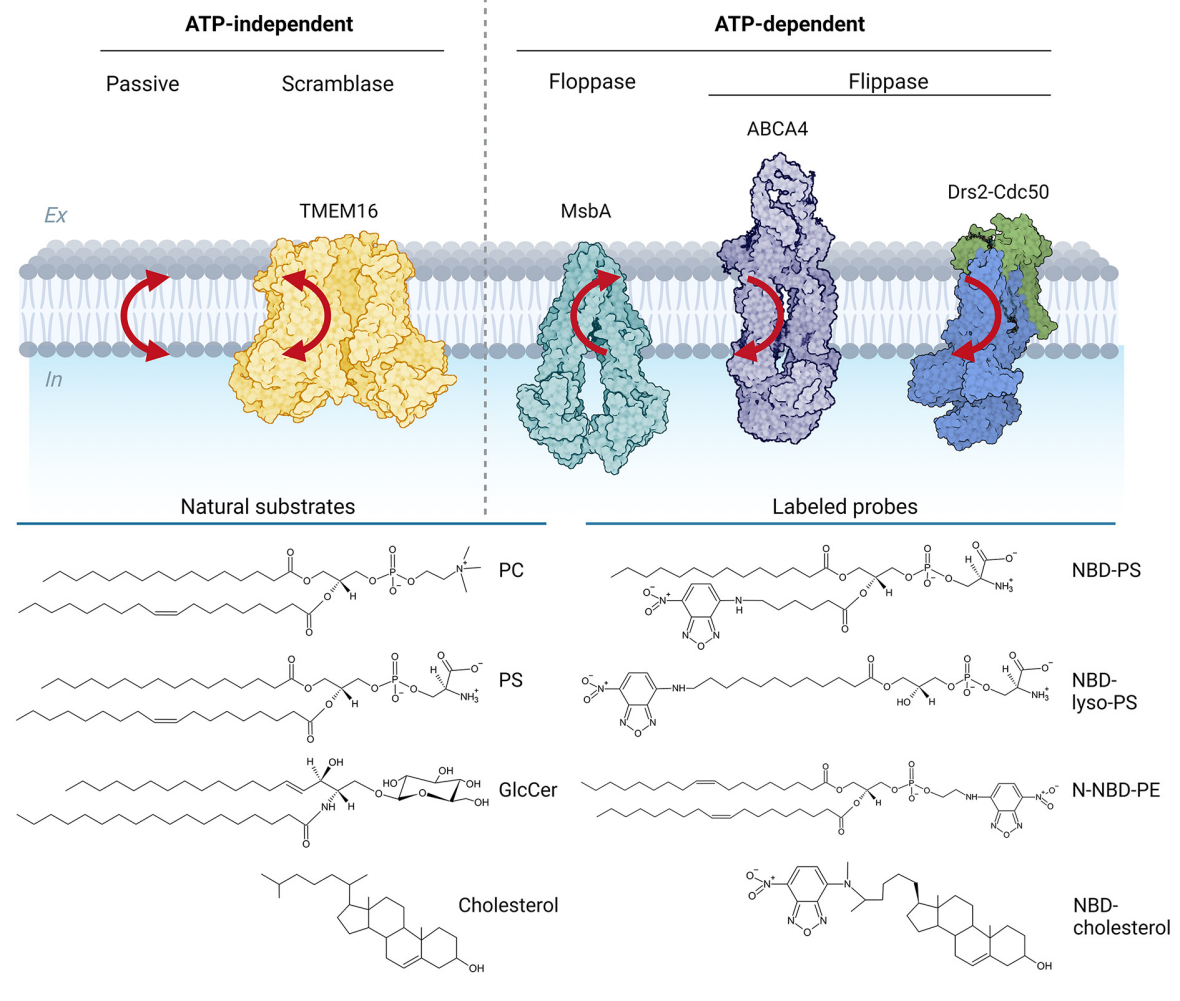
Transbilayer lipid movement in membranes (**A**) There are ATP-dependent and ATP-independent lipid translocation processes. ATP-independent transport includes flip-flop and scramblase-mediated transport, by, e.g. the Ca^2+^-activated scramblase TMEM16 (PDB code: 6P46). ATP-dependent transporters include floppases and flippases that move lipids against a chemical gradient at the expense of ATP. Lipid floppases move lipids from the cytosol to the extracellular/luminal leaflet and are represented by the ABC transporter MsbA (PDB code: 5TV4). Lipid flippases move lipids from the extracellular/luminal leaflet to the cytosolic leaflet and include P4-ATPases (e.g. Drs2-Cdc50, PDB code: 6ROJ) and some ABC transporters (e.g. ABCA4, PDB code: 7LKP). The direction of lipid movement is indicated by red arrows. (**B**) Chemical structures of some lipid substrates are shown, including phosphatidylcholine (PC), phosphatidylserine (PS), glucosylceramide (GlcCer) and cholesterol and labeled probe lipids, represented by nitrobenzoxadiazol (NBD) labeled lipids.

There are three types of lipid transporters: scramblases, flippases, and floppases (Figure 1). Scramblases allow bidirectional transport and work by moving lipids down their concentration gradient at an impressive rate of over 10^4^ lipids per second [7], thereby rapidly randomizing the distribution of lipids across the bilayer. They can be constitutively active or regulated by physiological stimuli, such as increase in intracellular Ca^2+^ or proteolytic cleavage [8–10]. In contrast, lipid flippases and floppases are transmembrane proteins that use the energy of ATP hydrolysis to transport specific lipids unidirectionally against their concentration gradient to the cytoplasmic or exoplasmic/luminal leaflet of cellular membranes, respectively, at a rate of approximately 10–100 lipids per second. The interplay between members of these two ATP-dependent primary transport systems is thought to be responsible for the maintenance of membrane lipid asymmetry. Membrane asymmetry is critical for cell viability and is involved in key cellular processes such as signaling events to initiate blood coagulation [11], phagocytes’ recognition of cells undergoing apoptosis and subsequent clearance by macrophages [12], and host–virus interactions where it might be important to ensure viral infection [13].

Recent advances in structural biology and computational biology have made it possible to capture the structures of several lipid transporters in multiple functional states (reviewed in [14–17]). With the increasing number of resolved structures of lipid transporters and the development of molecular dynamics simulations [18–20], we now have a more comprehensive understanding of how these transporters may move lipids. At the same time, advances in the field of mass spectroscopy and quantitative lipidomics have paved the way for the study of intact membrane proteins in association with bound lipids, thereby facilitating the identification of potential lipid substrates and cofactors [21]. However, experimental validation is needed to confirm and refine the current models, especially regarding molecular details such as the precise stoichiometry of coupling ATP hydrolysis to substrate transport and regulation under (semi-) physiologic conditions. In this review, we focus on the current progress and challenges in reconstituting ATP-dependent primary lipid transporters into large unilamellar vesicles and in characterizing their substrate specificity and regulation.

## ATP-dependent primary lipid transporters

ATP-dependent primary lipid transporters are essential membrane proteins that belong to either the P-type ATPase family or the ATP-binding cassette (ABC) transporter family. The P-type ATPase family can be divided into five major evolutionarily related subfamilies (P1-P5), each with distinct transport specificities. Among these subfamilies, P4-ATPases function as lipid flippases and are found exclusively in eukaryotes. The P-type ATPases have a similar structure, consisting of a membrane-spanning (M) domain and three cytosolic domains. These include an autocatalytic phosphorylation (P) domain, a nucleotide binding (N) domain, and an actuator (A) domain, which are responsible for the structural transfer of free energy release, derived from ATP hydrolysis. The amino and carboxyl termini of P-type ATPases face the cytosol, vary in length, and often accommodate regulatory domains or motifs. During substrate transport, P4-ATPases undergo conformational changes and the auto-phosphorylating and dephosphorylating of a conserved aspartate residue within a conserved signature sequence, hence the name ‘P-type’. P4-ATPases typically form heterodimeric complexes with an accessory subunit known as cell division control protein 50 (Cdc50), which are integral membrane proteins glycosylated in their exoplasmic domain. The interaction between Cdc50 proteins and individual P4-ATPases is crucial for endoplasmic reticulum exit and transporter activity [22–25].

Members of the ABC transporter superfamily include ATP-dependent lipid transporters that function either as flippases, floppases or possibly as lipid importers/exporters. These transporters are found in bacteria, archaea and eukaryotes, and the wide diversity within this family is exemplified by differences in structure, mechanistic features, and substrates transported [17,26–28]. All ABC transporters share a core topology, consisting of two transmembrane domains that facilitate substrate translocation across the membrane and two cytoplasmic nucleotide-binding domains. In bacteria, each of the four domains is either a distinct subunit or a combination of fused nucleotide-binding domains and/or transmembrane domains. In eukaryotes, these domains are organized either as full transporters or as ‘half-transporters’ with either identical (homodimeric) or different (heterodimeric) halves. Some ABC transporters use phospholipids as substrates, while others facilitate the transport of sterols. In addition, many of these transporters have been implicated in drug resistance and in the transport of other amphipathic and hydrophobic molecules, pointing to a close relationship between the mechanisms of drug extrusion and lipid translocation across membranes [28,29].

The mechanism by which P4-ATPases and ABC transporters flip-flop lipids is still not fully understood. The classical transport model for P4-ATPases is known as the ‘credit card model’ as an analogy to a credit card being swiped through a reader terminal by only contacting with the magnetic stripe. This model proposes that as phospholipids cross the membrane, only their headgroups gain access to the central cavity, while the hydrophobic hydrocarbon tails remain immersed in the hydrophobic core of the bilayer [30]. Recent structural data obtained from cryo-EM and X-ray crystallography support this model and suggest that the lipid transport pathway in P4-ATPases contains additional elements from three previously described models [reviewed in 14]. For ABC transporters, the ‘alternating access model’, which typically describes most primary and secondary transporters, suggests that the pump alternates between three major conformational states: inward-open, occluded, and outward-open. First, the inward-open transporter allows the substrate to enter a central cavity, leading to the occlusion of the transporter. Then, through an additional conformational change, the transporter opens towards the exoplasmic side to finally release its substrate. In contrast with P4-ATPases, the central cavity of some ABC transporters is wider and can accommodate a complete lipid molecule. This has been shown by the structural characterization of the PC translocator ABCB4 localized in the canalicular membrane of the hepatocyte [31], the lysosomal transporter ABCB9 [32] that acts as a peptide translocator and phosphatidylserine (PS) floppase and the bacterial ABC transporter MsbA that exports lipopolysaccharides from the inner surface to the outer surface of the inner bacterial membrane [33]. However, in line with the ‘credit card model’, it may be sufficient for only the lipid head group to enter the central cavity, as later suggested for ABCB4 [34].

Based on recent advances in molecular dynamics simulations and structure determination of eukaryotic and bacterial ABC transporters, new transport models have been proposed that deviate from the canonical alternating access mechanism. Examples include the ‘outward-only’ mechanism with outward-occluded and outward-open conformations, proposed for lipid-linked oligosaccharide flopping by PglK, which does not invoke an inward-facing cavity to interact with the substrate [35]. This bacterial ABC transporter catalyzes the translocation of lipid-linked oligosaccharides, which are essential for the bacterial protein glycosylation machinery [36]. Similarly, it has been proposed that ABCA1 does not form an inward-facing transmembrane cavity, which appears to be required for the alternating access model [37]. This mammalian transporter effluxes cholesterol and phospholipids to lipid free apolipoprotein A-I, when the latter binds to the membrane bilayer and generates nascent high-density lipoprotein [38,39]. In addition, based on simulations, a recent study proposes that ABCA1 extracts phospholipids from the outer leaflet of a model plasma membrane into the outward-facing cavity. The lipid then diffuses towards an elongated hydrophobic tunnel and is finally extruded out of the transporter [40]. In this case, the transporter would not promote transbilayer movement, but instead the release of the lipid out of the membrane. To understand the precise mechanisms of lipid translocation by the various transporters, further high-resolution structures with bound lipid substrates in combination with biochemical assays are required.

## Studying lipid transporter activities: from cell-based assays toward functional reconstitution

Although ATP-driven lipid transporters have been extensively studied, much of our current understanding has come from cell-based studies. The first discovery of ATP-dependent lipid flippase activity dates back to the 1980s, based on pioneering work using spin-labeled lipid probes [41]. Subsequently, lipid uptake assays using exogenously applied fluorescent-labeled lipids were developed [42–44] and have since been widely used to study ATP-driven lipid transporters in various organisms, including mammalian cells, plants, parasites, and yeast. In recent years, heterologous expression in *Saccharomyces cerevisiae* has become an increasingly popular tool for the functional analysis of candidate lipid flippases. This is due to the ease of handling and the availability of numerous deletion mutants [45–48].

**Figure 2:**
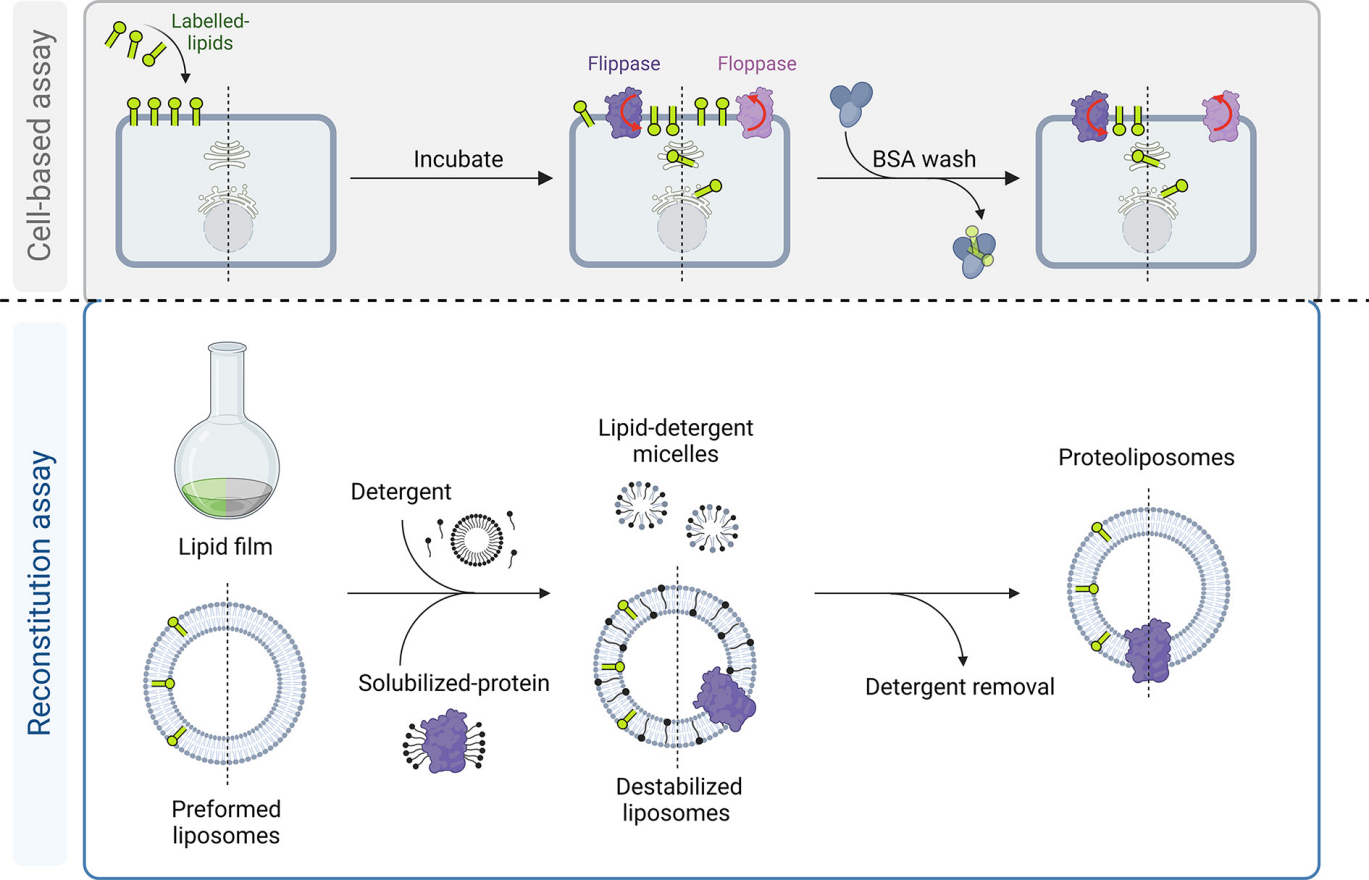
Schematic representation of cell-based and reconstitution approaches for ATP-dependent lipid transporter analysis Cell-based assays use short-chained labeled lipids (in green) that incorporate into the plasma membrane of cells expressing the transporter of interest. The lipid probes employed are typically either potential substrates of flippases (dark purple) or lipid pre-cursors that can freely cross the plasma membrane and after their intracellular metabolic conversion serve as substrate of floppases (light purple). Back-exchange with albumin (BSA, light blue) is then performed to remove the non-internalized fraction of probes or the flopped newly synthesized labeled probes, respectively. For lipid transport studies *in vitro*, ATP-dependent lipid transporters are reconstituted from lipid-protein-detergent micelles or detergent-saturated liposomes. Subsequent detergent removal results in the formation of proteoliposomes.

Cell-based assays to study lipid transport are typically based on short-chained nitrobenzoxadiazol (NBD)-labeled lipids (Figure 2, top panel). The flippase-mediated transport of these probes is usually monitored by extracting the residual fraction of analogues not transported across the membrane with bovine serum albumin (BSA). Since BSA extracts all analogues from the exoplasmic monolayer of the plasma membrane, the inaccessible fraction reflects analogues that have been redistributed across the plasma membrane into cells [49,50]. Alternatively, NBD-labeled lipids on the exoplamic monolayer can be selectively destroyed with the water-soluble quencher dithionite, followed by quantification of the intracellular fluorescence [56,135]. To study floppase-mediated transport across the plasma membrane, cells can be incubated with lipid precursors. Under these conditions, for example, short-chain NBD-phosphatidic acid (NBD-PA) is partially converted into NBD-diacylglycerol, which rapidly crosses the plasma membrane and becomes available for the intracellular synthesis of NBD-PC and NBD-phosphatidylethanolamine (NBD-PE) [51–53]. The continuous incubation of the cells in the presence of BSA allows then monitoring of the transport of the newly synthesized analogues to the cell surface by lipid analysis.

Such cell-based studies conducted on P4-ATPases have contributed significantly to our understanding of their lipid specificity (Figure 2, upper panel). Although originally classified as aminophospholipid flippases, individual members of this protein family exhibit distinct substrate preferences and can transport a diverse range of lipid substrates. In general, P4-ATPases can be categorized into three groups: those that preferentially transport PS and to a lesser extent PE, those that preferentially transport PC and PE, and those with a wide range of lipid substrates, including sphingolipids, lysophospholipids, and synthetic alkyl phospholipids [54]. Similarly, cell-based studies have helped to elucidate the lipid substrate specificities of some ABC transporters. For example, studies using short-chain lipids have revealed that human ABCB4 exhibits specificity for PC, whereas the closely related multidrug transporter ABCB1 translocates a variety of short-chain lipids [52,53,55] including platelet-activating factor, also known as acetyl-glyceryl-ether-phosphorylcholine [56]. Studies using (radiolabeled) cholesterol and phospholipids have demonstrated the role of ABCA1 in mediating the efflux of cholesterol and phospholipids such as PC [57–60].

A major challenge in cell-based studies is that cells typically express multiple lipid transporters, some of which have overlapping transport functions. In addition, the expression pattern of these transporters as well as the amount of lipid substrate transported can vary depending on the cell type and the specific expression approach used. This variability may explain, for example, the conflicting substrate specificities reported for the human P4-ATPase ATP8B1. Although initially characterized as a translocase for aminophospholipids [61–63] and potentially for cardiolipin [64,65], other studies found that ATP8B1 translocates PC rather than PS [66] or in some cases, might be inactive [67] upon overexpression in cell lines with low endogenous phospholipid flippase activity. Furthermore, cell-based assays typically monitor the lipid internalization at the plasma membrane, which is influenced by endocytic and exocytic membrane trafficking events. It is therefore important to either minimize these processes, e.g., by lowering the temperature, or to quantify them by an independent measurement before drawing conclusions from the experimental data.

Advances in expression and purification techniques have allowed the biochemical characterization of an increasing number of lipid transporters at the solubilized stage and after reconstitution into proteoliposomes. This approach provides a well-defined system that allows investigating defined conditions outside the complex cellular environment. Parameters, such as lipids, salt concentration, presence of substrates, and cofactors, can be tested and then tailored to meet the requirements of the protein under study. Liposomal reconstitution offers in addition a two-compartment system that permits tracking of the transport of substrates. First, the transporters are usually overexpressed in a suitable expression host and then solubilized from the membrane using a mild detergent, followed by purification and concentration. The choice of detergent is primarily based on empirical testing, and therefore, detergent screenings are a common practice for ATP-dependent lipid transporters [68]. The purified transporters are then reconstituted into large liposomes, also referred to as large unilamellar vesicles, with diameters of 100–200 nm. However, reconstitution remains a challenging task due to difficulties in purifying membrane proteins to sufficient yields, the ‘trial and error’ approach required to successfully reconstitute active transporters, and the potential need for co-reconstitution of accessory proteins [69,70].

Several methods have been developed for the rapid and effective reconstitution of membrane proteins into lipid membranes. The most commonly used method for the preparation of proteoliposomes is based on detergent-mediated reconstitution [71–73]. In this approach, the solubilized membrane protein is supplemented with an excess of either preformed large unilamellar vesicles or a lipid suspension, together with detergent, resulting in a mixture of lipid-protein-detergent and lipid-detergent micelles. The detergent is then removed, resulting in the progressive formation of a vesicle bilayer, thereby embedding the protein (Figure 2, lower panel). ATP-dependent lipid transporters are typically reconstituted using detergents, with octyl glucoside and Triton X-100 being the most frequently used (Table 1). Of note, the use of C_12_E_9_ for reconstitution of a P4-ATPase resulted in vesicles that were too leaky for lipid transport studies [74]. The method used to remove the detergent depends on the physicochemical properties of the detergent employed [73]. To reconstitute the ATP-dependent transporters summarized in this review (Table 1), dialysis followed by gel filtration was used to remove octyl glucoside, whereas hydrophobic beads (Bio-beads) were used to remove Triton X-100 and polyoxyethylene detergents. After proteoliposome formation, a centrifugation step is sometimes added to concentrate the proteoliposomes [32,75,76] and/or to remove unincorporated protein from the proteoliposomes [18,77]. Membrane proteins reconstituted in liposomal model membrane systems have either the cytosolic side (inside-out orientation) or the extracellular side (outside-out orientation) exposed. ATP-dependent transporters appear to prefer an inside-out orientation in the vesicle, due to bulky domains and the curvature of the vesicle [78] but the exact protein orientation is hardly predictable, which limits the quantitative and functional analysis of vesicle preparations [79,80].

**Table 1:** ATP-dependent lipid transporters reconstituted and analysed in liposomal systems

Lipid transporter	Method^1^	Liposomes^2^	Lipid tested^3^	Amplitude^4, 5^	Reference
**Mammalian P4-ATPases**					
ATP8A2-CDC50A	Lipid suspension, OG, dialysis Dithionite quenching	PC	2.5 wt% NBD-PS, NBD-PE, NBD-PC	∼9% NBD-PS, ∼1% NBD-PE	[86,24,123]
ATP8A2-CDC50A	Lipid suspension, OG, dialysis Electrophysiology in SSM	PC/PS (9:1 or 9.9:0.1) PC/PE (9:1 or 5:5) PC	PS, PE, PC	PS, PE	[18,77]
ATP8A1-CDC50A	Lipid suspension, OG, dialysis Dithionite quenching	PC	2.5 wt% NBD-PS, NBD-PE	∼12% NBD-PS, ∼8% NBD-PE	[124]
ATP11A-CDC50A	Lipid suspension, OG, dialysis Dithionite quenching	PC	2.5 wt% NBD-PE, NBD-PS	∼5% NBD-PS, ∼1% NBD-PE	[88]
ATP11B- CDC50A	”	”	”	∼4% NBD-PS, ∼2% NBD-PE	[88]
ATP11C-CDC50A	”	”	”	∼7% NBD-PS, ∼3% NBD-PE	[88]
Mg^2+^ ATPase (ATP11C)^6^	Lipid suspension, TX-100, Bio-Beads Ascorbate quenching	PC PC/PI (9.8:0.2)	1 mol% SL-PS, SL-PE, SL-PC	∼13% SL-PS (both liposomes) ∼7% SL-PE (only in PC:PI)	[125]
ATP8B1-CDC50A/B	Lipid suspension, TX-100, Bio-Beads Dithionite quenching	Brain lipids	0.5 mol% NBD-PS, NBD-PC	∼3% NBD-PS	[126]
***Saccharomyces cerevisiae* P4-ATPases**					
Drs2-Cdc50	Lipid suspension, C_12_E_9_, Bio-Beads Dithionite quenching	PC	1 wt% NBD-PS, NBD-PC, NBD SM	∼4% NBD-PS	[87]
Drs2-Cdc50	Lipid suspension, C_12_E_8_, Bio-Beads Dithionite quenching	PC w/o PI(4)P	2.5 mol% NBD-PS, NBD-PC	NBD-PS FL: ∼9% w/PI(4)P, ∼1.5% w/o PI(4)P Truncated: ∼7% w/PI(4)P),∼14% w/o PI(4)Pz	[74]
**Mammalian ABC transporters**					
ABCA1	Preformed liposomes, CHAPS, dialysis Dithionite and collisional quenching	PG PC	0.6 mol% of NBD-PC, NBD-PS, NBD-PE, NBD-PG, NBD-SM	∼8.5% NBD-PC, ∼5.5% NBD-PS, ∼4.5% NBD-SM (in PG); ∼1% NBD-PC, ∼3% NBD-PS, ∼1% NBD-SM (in PC)	[82]
ABCA4	”	”	”	∼7% NBD-PE (in PG) ∼3% NBD-PE (in PC)	[82]
ABCA7	”	PG	0.6 mol% of NBD-PC, NBD-PS	∼9% NBD-PS, 4% NBD-PC	[82]
ABCB1 (Pgp, MDR1)	Lipid suspension, CHAPS, filtration chromatography Dithionite quenching	PC	0.3 wt% NBD-PC, NBD-PE, NBD-PS, NBD-SM, N-NBD-PE	3.4%-0.6%, from high to low: NBD-SM, NBD-PC, N-NBD-PE, NBD-PS, NBD-PC, NBD-PE, NBD-PS	[78]
ABCB1 (Pgp, MDR1)	Lipid suspension, CHAPS, gel filtration chromatography Dithionite quenching	PC	0.3 wt% NBD-PC, NBD-SM, NBD-GlcCer, NBD-GalCer, NBD-LacCer, NBD-Cer	3.1%-0.48% from high to low: NBD-SM, NBD-GalCer; NBD-GlcCer, NBD-PC, NBD-LacCer	[101]
ABCB4	Preformed liposomes, TX-100, Bio-Beads Dithionite quenching	Liver lipids/CL (4:1)	0.3 mol% NBD-PC, NBD-PE	∼6.5% NBD-PC	[31]
ABCB9 (TAPL)	Preformed liposomes, DM, Bio-Beads Dithionite quenching	*E.coli* lipids/PC (1:1)	0.5 mol% NBD-PS, NBD-PG, NBD-PE, NBD-CL	∼2.6% NBD-PS	[32]
ABCC1 (MRP1)	Preformed liposomes, CHAPS, gel filtration chromatography Dithionite quenching	Asolectin	0.3 wt% NBD-PC	∼10% NBD-PC	[75]
ABCG5-ABCG8 (G5-G8)	Lipid suspension, OG, Dialysis Donor-acceptor assay	PC:PE:PS:PI:SM:CL Only PC	[^3^H]CL, [^3^H]ent-CL, [^3^H]sitosterol [^3^H] cholesteryl oleate, [^3^H]PC	∼10% [^3^H]CL, sitosterol	[84,85]
**Fungal ABC transporters**					
Cdr1	Lipid suspension, TX-100, Bio-Beads Dithionite quenching	PC	0.3 mol% NBD-PC, NBD-PE, NBD-PS, N-NBD-PE	∼9% NBD-PE, 7.6% NBD-PC, 5.3% NBD-PS and 5% N-NBD-PE	[127]
**Bacterial ABC transporters**					
LmrA	Preformed liposomes, DDM, Bio-Beads Donor-acceptor assay, collisional quenching	*L. Lactis* lipids	3 mol% NBD-PE, NBD-PC; 6 mol% N-Rho-PE	∼20% NBD-PE	[83]
MsbA	Lipid suspension, OG, gel filtration chromatography Dithionite quenching	*E.coli* lipids *E.coli* lipids/PC (1:1 or 7:3)	0.3 mol% NBD-PC, NBD-PE, NBD-PS, NBD-PG, NBD-SM, NBD-GlcCer, NBD-LacCer; N-NBD-PS, N-NBD-PE	∼7.7% NBD-PE, 4.7% NBD-PG, 4.3% NBD-GlcCer, 4% NBD-PS, 3.6% NBD-PC, 2.6% NBD-SM, 2.5% N-NBD-PE, 1.6% N-NBD-PS	[128]
MsbA	Preformed liposomes, TX-100, Bio-Beads Biotin - fluorescent avidin Dithionite quenching	*E.coli* lipids/PC (3:1)	0.12 wt% N-Biotin-PE, 0.5 wt% Biotin-Lipid-A or 0.012 wt% N-NBD-PE	N-biotin-PE: ∼2-3% (+ATP or +∆pH) and ∼6-7% (+ATP + ∆pH); similar for N-NBD-PE Biotin-Lipid-A: ∼5% (+ATP, + or - ∆pH)	[76]
Pglk	Preformed liposomes, TX-100, Bio-Beads Radiolabelling via glycosyltransferase	*E.coli* lipids/PC (3:1)	∼0.08 mol% tLLO	∼25% tLLO	[35]

Abbreviations: CL, cholesterol; DDM, n-Dodecyl β-D-maltoside; DM, n-decyl-β-D-maltopyranoside; FL, full-length; GalCer, galactosylceramide; GlcCer, glucosylceramide; LacCer, lactosylceramide; MDR1, multidrug resistance protein 1; MRP1, multidrug resistance protein 1; NBD, nitrobenzoxadiazol; N-Rho-PE, head-labeled rhodamine phosphatidylethanolamine; OG, n-octyl-β-D-glucopyranoside; PC, phosphatidylcholine; PE, phosphoethanolamine; PG, phosphatidylglycerol; Pgp, P-glycoprotein multidrug transporter; PI(4)P, phosphatidylinositol-4-phosphate; PI, phosphatidylinositol; PS, phosphatidylserine; SL, spin-labeled; SM, sphingomyelin; SSM, solid supported membranes; TAPL, transporter associated with antigen; tLLO, trisaccharide lipid-linked-oligosaccharide.

1 "Method” refers to the reconstitution approach (starting material: Lipid suspension or preformed liposomes, also known as large unilamellar vesicles; detergent used for reconstitution; detergent removal; method for monitoring lipid transport/transfer).

2 Lipid composition of lipososomes used for reconstitution.

3 The NBD-group is attached to an acyl chain (NBD-) or headgroup (N-NBD-).

4 Amplitude = % of lipids transported.

5 Amplitude range indicates differences depending on substrate tested.

6 The Mg^2+^-ATPase activity is likely based on ATP11C [129].

## Assaying of lipid transporter activities in proteoliposomes

**Figure 3:**
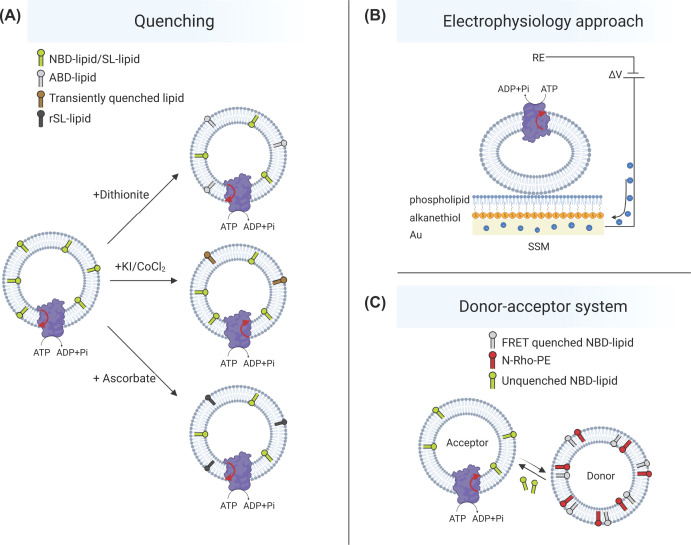
Schematic representation of the approaches to measure lipid transport in liposomes (**A**) Quenching assays use fluorescent-labeled lipids, e.g. NBD-lipids (green). In the dithionite assay, the membrane-impermeable quencher dithionite reduces the NBD group into a nonfluorescent derivative (ABD-lipid, gray). In the collisional quenching assay, potassium iodide (KI) or cobalt chloride (CoCl_2_) generate a non-radiative transition to the ground state of the NBD-group (Transiently quenched lipid, brown). In case of spin-labeled lipids, chemical reduction by aqueous ascorbate is used to generate reduced spin-label (rSL-lipid, black). (**B**) In the electrophysiological method, proteoliposomes are adsorbed on a solid supported membrane and subjected to ATP activation. Upon transport of charged lipids, a current signal is detected (adapted from [77]). RE, reference electrode; ΔV, potential difference; SSM, solid supported membrane. (**C**) Donor-acceptor approaches require two sets of liposomes, e.g., donor vesicles containing a FRET-pair, here an NBD-lipid together with a headgroup labeled N-rhodamine lipid such as PE (N-Rho-PE) and acceptor proteoliposomes without fluorescent lipids. Exchange and translocation of the NBD-lipid can be quantified by measuring the decrease of the energy transfer between the NBD lipid and the N-Rho-PE or by measuring NBD-fluorescence after separating donor from acceptor vesicles.

Various assays are available for measuring ATP-dependent lipid transporter activities in proteoliposomes (Figure 3 and Table 1). A common method is to use labeled lipid reporter molecules carrying a fluorescent group (Figure 1, lower panel) or, less commonly, a spin-labeled group. The transbilayer transport of these probes can be monitored over time by chemical modification of the (non-)translocated lipid probes with membrane-impermeable reagents such as dithionite for e.g. NBD-labeled lipids and ascorbate for spin-labeled lipids. Another approach to assess lipid transport based on fluorescent-labeled lipids is the collisional quenching assay (Figure 3A). Collisional quenching utilizes membrane-impermeable contact quenchers such as iodide or cobalt that generate a nonradiative transition to the ground state of the fluorescent group upon contact [81,82].

Isotopic modification of lipids has also been utilized to assess lipid transport in liposomal systems. For example, the flippase activity of the bacterial ABC transporter PglK was tested using a truncated form of its natural substrate (GlcGalNAc_5_Bac-PP-undecaprenyl). The method is based on the use of a soluble glucosyltransferase acting in the presence of an excess of nucleotide-bound radiolabeled sugars to convert the truncated substrate upon its appearance in the outer leaflet of the liposome. The change in radioactivity over time provides a quantitative measure of the rate at which PglK flips the truncated substrate ([35]; Table 1).

Recently, a novel approach has been employed to study the energetics of lipid transport by the ABC transporter MsbA [76]. This transporter accepts as substrate PE carrying a biotin moiety covalently linked to the headgroup. This allows measuring the amount of accessible biotin-labeled lipid in proteoliposomes from the fluorescence emission of fluorescence-tagged avidin, initially bound to a quencher, which becomes displaced when avidin binds to the biotin moiety.

When studying charged lipid substrates, such as PS, electrophysiological measurements can be a useful approach. In this method, proteoliposomes are loaded onto a solid-supported membrane and a transient electrical current is measured upon the addition of ATP. As an ATP-dependent transporter moves charged lipids across the membrane, the capacitive coupling between the proteoliposomes and the solid-supported membrane allows the measurement of the resulting current transient [18,77] (Figure 3B). This current can provide two types of information: firstly, the decay time constant, i.e., the rate at which charges are moved, can be calculated by fitting the decay of the current signal with a first-order exponential function. Secondly, the amplitude of the current signals, which represents the total number of charges moved, can be calculated by integrating the current signals. These parameters can then be used to assess the activity of the transporter and the kinetics of the lipid transfer process. Notably, this assay does not require lipid probes and can be used with endogenous lipids.

Another type of assay is based on the use of donor vesicles that are added to the proteoliposomes. In this approach, donor liposomes are prepared containing, e.g. short-chain NBD-lipids and rhodamine-labeled PE (N-Rho-PE), which cannot translocate from the donor liposomes to the proteoliposomes. During the exchange and translocation of the NBD-lipid, a decrease in the energy transfer between the NBD-lipid and the N-Rho-PE occurs, resulting in an increase in NBD fluorescence and enabling continuous measurement of lipid translocation (Figure 3C). This method has been used to assess lipid transport by an ABC transporter [83]. Studies conducted by Wang and co-workers [84,85] have shown that the donor vesicle approach is also an effective method for observing the transfer of radiolabeled sterols. The results of donor–acceptor vesicle approach, however, should be interpreted with care. Since this method involves the transfer of lipids between vesicles, the assay may measure the ability of the transporter to insert or extrude lipids into or out of the membrane rather than to transport them across the bilayer.

## Insights from *in vitro* studies

To date, only a small number of P4-ATPases and ABC transporters have been successfully reconstituted (see Table 1 and references therein). In most cases, the assignment of lipid translocation activity has been based on the use of fluorescent lipid probes, with only a few studies attempting to measure the transport of natural lipids. Nevertheless, the reconstitution experiments performed so far provide the best evidence that these transporters directly catalyze lipid transport.

## P4-ATPases: lipid flippases with different substrate specificities

One of the first purified P4-ATPases studied upon reconstitution was ATP8A2, a P4-ATPase present in the disc membranes of rod and cone photoreceptors. Upon reconstitution into chemically-defined liposomes, the purified enzyme was found to flip fluorescent-labeled PS [86]. Similarly, reconstitution of the yeast P4-ATPase Drs2 showed that this enzyme catalyzes PS transport [87]. Substrate competition assays have been used here to verify that natural lipids are indeed substrates. The natural lipid substrate competes with the fluorescent probe, resulting in a decrease in the transport activity toward the lipid probe. Such inhibition has been demonstrated, for example, for the P4-ATPases ATP8A2 [86] and ATP11A, B, C [88]. Further biochemical characterization of P4-ATPases at the solubilized level [89–91] has shown that the lipid headgroup is the key structural element for substrate recognition by P4-ATPases. However, the structure of the lipid tail may influence the correct positioning of the glycerol backbone and thereby affect the recognition of mono- and di-acyl lipids.

Another important finding is the identification of phosphoinositides as activators of some P4-ATPases, as shown for the P4-ATPases Drs2 [74,92–94], ATP8B1 [95], and ATP2 [96]. The C-terminal extension of Drs2, together with the transmembrane domain 10, contains amino acids that bind phosphatidylinositol-4-phosphate [94], and the presence of this phosphoinositide stimulates the flippase activity of the enzyme in proteoliposomes [74]. This finding suggests that phosphoinositides may relieve the autoinhibition imposed by the C-terminus of Drs2 that can bind in between the P- and the N- domains, thereby restricting potential domain movements required for pumping [93,94]. A non-exclusive possibility is that phosphoinositides act as lipid cofactors, for example, by potentially regulating substrate access to lipid transport pathway in the protein by inducing conformational changes in the transmembrane domain. This notion is supported by the observation that the activity of the purified human P4-ATPase ATP8B1 is stimulated by phosphoinositides only when the C-terminus is removed [95]. The N-terminus of Drs2 and ATP8B1 also seem to participate in the autoinhibition of these transporters and together with the C-terminus might have a synergistic effect on autoinhibition [92,95]. In addition, some P4-ATPases share the (G/A)(Y/F)AFS motif at the C-terminus, which is involved in autoinhibition through interaction with the N-domain [48,95,96]. Thus, regulation by the C-terminus may be a common feature of this transporter family. Furthermore, the activity of some P4-ATPase in cells is regulated by interacting proteins such as kinases and thus, phosphorylation [97–99] and small GTP-binding proteins [100] that might participate in autoinhibition release. However, it has not yet been possible to recapitulate this requirement *in vitro*.

## ABC transporters: a family of exporters and importers

One of the first ABC transporters to be reconstituted for analysis of lipid transport activity was the human multidrug resistance transporter ABCB1 [78]. After reconstitution into chemically-defined liposomes, it was shown to act as a floppase for a variety of fluorescent-labeled phospholipids and sphingolipids [78,101], including simple glycolipids. Similarly, the ABCB4 and the glutathione-dependent multidrug transporter ABCC1 were shown to transport fluorescent-labeled PC after reconstitution into proteoliposomes [31,75], while ABCA1 and ABCA7 transported fluorescent-labeled PC, PS, and SM with a preference for PC and PS, respectively [82]. In contrast, ABCA4 was shown to function as an importer flipping fluorescent-labeled PE in liposomes and radiolabeled N-retinylidene-PE in photoreceptor disc membranes and proteoliposomes [82,102]. The ABC transporter PglK from *Campylobacter jejuni* catalyzed the translocation of lipid-linked oligosaccharides *in vivo* [36] and upon reconstitution into liposomes, PglK translocated an analog of the natural lipid-linked oligosaccharide [35]. Functional reconstitution allowed also demonstration of the stereospecific sterol transfer activity of co-purified ABCG5/G8 [84]. Thus, similar to P4-ATPases, individual ABC transporters differ in their substrate specificities. Some ABC transporters may have a dual substrate specificity, as recently uncovered for the lysosomal ABC transporter ABCB9 [32]. In addition to its role as a peptide transporter, ABCB9 was found to transport fluorescent-labeled PS after reconstitution into proteoliposomes.

As much of the evidence is based on the transport of fluorescent-labeled lipids, the selectivity for labeled substrates may not necessarily apply to the natural substrates in each case. In some instances, substrate competition data provide evidence that natural lipids act as substrates. For example, the transport of fluorescent-labeled PE by ABCA4 is inhibited by the presence of PE, whereas the transport activity of ABCA1 for fluorescent-labeled PC is suppressed by PC [82]. In the case of MsbA, the presence of physiologically relevant substrates such as lipid A was found to inhibit the translocation of PE probes in proteoliposomes [101]. Interestingly, a new twist to the story came when recent work identified the requirement of a chemical proton gradient for the transport of PE by MsbA ([76]; Table 1). Notably, flopping of the large hexa-acylated Lipid-A required only ATP, pointing out that the energetic requirements of MsbA as a lipid transporter are substrate dependent. Future studies will need to define if this property is shared by other members of the ABC transporter and possibly, of the P4-ATPase families.

Reconstitution studies have also been instrumental in elucidating the dependence of the ABC transporters on specific lipids for their activity. In particular, phospholipids and sterols have been shown to influence the activity of several ABC transporters involved in lipid translocation. One example is the yeast ABC transporter Aus1, which localizes primarily to the plasma membrane and, together with Pdr11, is required for sterol uptake under anaerobic conditions. Upon reconstitution into proteoliposomes, the ATPase activity of Aus1 was specifically stimulated by PS in a stereoselective manner [103]. Presence of cholesterol in liposomes reconstituted with the mammalian ABC transporters ABCA1 and ABCA4 reduced the transport of fluorescent-labeled PC and PE, respectively [82]. However, it is important to note that in these experiments it is not possible to distinguish whether lipids modulate the protein indirectly by changing the properties of the lipid membrane or by acting as co-factors.

## Current limitations and future avenues

Despite their widespread use as a model membrane system, proteoliposomes have some limitations. Liposomes are closed bilayer structures of small dimensions with high curvature (diameter 100–200 nm). Therefore, unidirectional lipid transport in these vesicles, even at small amplitudes, can lead to the accumulation of bilayer stress [104]. This mechanical stress could feedback to inhibit transporter activity, indicating substrate saturation of the recipient leaflet [105]. It is therefore crucial to conduct careful controls to ensure that changes in the signal can be attributed to transporter activity. These might include experiments carried out in the presence of non-hydrolyzable ATP derivatives, inhibitors, in the absence of either ATP or Mg^2+^ and/or experiments carried out on reconstituted catalytically inactive mutants of the transporter under investigation. Another important aspect of liposome reconstitution is ensuring the absence of leakiness, which can be caused by residual detergent [73]. To detect potential leakiness in reconstituted liposomes, various approaches have been employed. One approach involves entrapping fluorescent probes, such as a fluorescent glucose analog, within liposomes, followed by dithionite quenching to assess leakage [106]. Alternatively, a self-quenching probe like calcein has been used, where leakage is monitored over time in a calcein leakage assay by recording changes in fluorescence [107].

**Figure 4:**
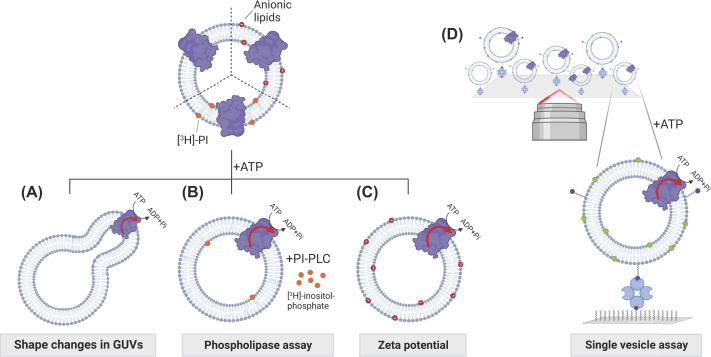
Promising assays and technologies to study lipid transport in model membrane systems (**A**) GUVs provide a valuable platform for functional analysis of reconstituted lipid transporters using light microscopy techniques without requiring labeled lipids. ATP-driven lipid translocation increases the proportion of total lipids in one monolayer of the vesicles, resulting in a shape change. (**B**) Phospholipases can be utilized to assess the transport of untagged lipids (adapted from [109]). (**C**) Zeta potential (ζ) measurements allow measuring the transport of anionic lipids. (**D**) Single vesicles tethered to a passivated glass surface and imaged on an individual basis with total internal reflection fluorescence (TIRF) microscopy permits parallel analysis of multiple parameters (physical size, tightness, unilamellarity, membrane protein content, and orientation) of individual proteoliposomes, thereby providing a detailed picture of the reconstituted membrane system (adapted from [120,121]).

As mentioned above, current cell-based and reconstitution assays commonly use lipid probes. These probes have a reporter group attached to a short fatty acid chain at the *sn*-2 position, while a long fatty acid chain is at the *sn-1* position (Figure 1, lower panel). However, a major drawback of these tagged probes is the substantial alterations of steric bulk and polarity with respect to the endogenous lipids, which may affect their recognition as substrates and affect the biophysical properties of membranes. In the case of ABC transporters that mediate the efflux of fluorescent dyes, the lipid probe may even be perceived as a drug analog rather than a lipid. In the future, new approaches will be needed to measure transbilayer lipid movement using non-labeled lipids. Promising steps have been taken for lipid scramblases, including shape-change visualization experiments in giant unilamellar vesicles (GUVs) [108], phospholipase-based assays [109], and detection of specific lipids from each membrane leaflet using lipid-binding protein domains [110] (Figure 4). GUVs, ranging in size from 1 to 100 µm, provide a valuable platform for functional analysis of lipid transporters using light microscopy techniques without requiring labeled lipids. In this setup, unidirectional lipid transport by energy-coupled flippases or floppases creates a mass imbalance between the two membrane leaflets, inducing shape changes in the giant vesicle (Figure 4A). However, the reconstitution of membrane transporters into GUVs has proved challenging due to the inherent instability of these large liposomes and the potentially unfavorable conditions used during the reconstitution process. As a result, successful application of this approach has been limited up to date to scramblases and the erythrocyte lipid flippase [108,111–113]. Furthermore, phospholipase-based assays have been used to detect lipid scrambling in proteoliposomes, and should also be useful for studying energy-coupled flippases and floppases. For example, Wang et al. [2018] used a phosphoinositol (PI)-specific phospholipase C and radiolabeled PI to detect phosphatidylinositol scrambling [109]. Matoba et al. [2020] investigated lipid scrambling in proteoliposomes by using a phosphoinositide 3-kinase and ATP to convert PI in the outer leaflet to phosphatidylinositol 3-phosphate (PI3P). The redistribution of this lipid was then analysed using a rapid freezing and freeze-fracture replica method together with a PI3P-recognizing protein domain [110].

Finally, techniques such as measuring the zeta potential using the mechanisms of electrophoresis [107], nuclear magnetic resonance spectroscopy [114] and small angle neutron scattering [115] have been tested to characterize liposomes. Zeta potential measurements exploit the correlation between the zeta potential and the surface charge density, which depends exclusively on the anionic lipid content in the outer leaflet of liposomes (Figure 4). Indeed, these measurements have been successfully employed to study the asymmetry of anionic lipids and their successful incorporation and stability in large unilamellar vesicles (LUVs) and GUVs [116–118]. However, the aforementioned approaches have not yet been applied to study lipid transport in reconstituted model membrane systems. Combined with advanced reconstitution protocols that enable the generation of proteoliposomes with an asymmetric lipid arrangement [119], such techniques may prove useful for measuring the transbilayer movement of natural lipids.

Another problem encountered in liposome reconstitution is sample heterogeneity, which can bias the quantitative analysis of lipid and protein content as well as protein activity. To overcome this challenge, a promising approach is to characterize the intrasample compositional variation at the single vesicle level. This approach is based on the visualization of single liposomes by microscopy, using fluorescently labeled lipids and reconstituted proteins with corresponding fluorescent labels. By using site-specific quenching of the fluorophores, it is possible to obtain a detailed characterization of the reconstituted proteoliposomes, including parameters such as size, tightness, lamellarity and the number of proteins per liposome as well as their orientation [120]. In addition, this approach allows functional characterisation of the reconstituted enzyme, providing insights into important features such as the transition between active and inactive states and the role of autoinhibitory domains in these two states, as well as intrinsic transport rates [121,122]. Watanabe et al. (2018) have recently developed an elegant method for the single-molecule analysis of membrane transporters using femtoliter chamber arrays. The femtoliter chambers are enclosed by asymmetrical membrane bilayers, with fluorescently labeled phospholipids present exclusively in one leaflet. These bilayers also contain individual membrane proteins, enabling the monitoring of lipid translocation events. This method offers a high level of sensitivity, allowing for detailed investigations into the mechanisms and kinetics of lipid transport processes.

## Summary

Lipid transporters are inherently difficult to study. On the one hand, this can be attributed to the difficulties in handling the integral membrane proteins and their delicacy in production, purification, and characterization of their assembly with membrane lipids. On the other hand, sophisticated techniques are required to study membrane proteins at the molecular level. Despite these challenges, researchers continue to make progress in understanding the mechanisms underlying transporter function and in developing new techniques for studying these critical cellular components. In this review, we included current published reports on ATP-dependent lipid transporters and their functional analysis using liposomal reconstitution. Such functional assays, coupled with structural analysis and computational studies, including both molecular dynamics and coarse-grained simulations, will be essential in unravelling the inner working of these important transporter classes. In addition, we highlighted recent progress in uncovering key molecular and regulatory mechanisms of these transporters, the role of which remains to be further defined by systematic and detailed studies in model membrane systems and *in vivo*.

## Abbreviations

ABC: ATP-binding cassette
BSA: bovine serum albumin
Cdc50: cell division control protein 50
cryo-EM: cryogenic electron microscopy
GlcCer: glucosylceramide
GUV: giant unilamellar vesicle
LUV: large unilamellar vesicle
N-Rho-PE: headgroup rhodamine-labeled phosphatidylethanolamine
NBD: nitrobenzoxadiazole
PA: phosphatidic acid
PC: phosphatidylcholine
PE: phosphatidylethanolamine
PI: phosphoinositol
PI3P: phosphatidylinositol 3-phosphate
PS: phosphatidylserine
TIRF: total internal reflection fluorescence

## References

[1] van Meer G., Voelker D.R. and Feigenson G.W. (2008) Membrane lipids: where they are and how they behave. Nat. Rev. Mol. Cell Biol. 9, 112–124 10.1038/nrm2330 18216768 PMC2642958

[2] Harayama T. and Riezman H. (2018) Understanding the diversity of membrane lipid composition. Nat. Rev. Mol. Cell Biol. 19, 281–296 10.1038/nrm.2017.138 29410529

[3] Cournia Z., Allen T.W., Andricioaei I., Antonny B., Baum D., Brannigan G. et al. (2015) Membrane Protein Structure, Function, and Dynamics: a Perspective from Experiments and Theory. J. Membr. Biol. 248, 611–640 10.1007/s00232-015-9802-0 26063070 PMC4515176

[4] Contreras F.-X., Sánchez-Magraner L., Alonso A. and Goñi F.M. (2010) Transbilayer (flip-flop) lipid motion and lipid scrambling in membranes. FEBS Lett. 584, 1779–1786 10.1016/j.febslet.2009.12.049 20043909

[5] Holthuis J.C.M. and Levine T.P. (2005) Lipid traffic: floppy drives and a superhighway. Nat. Rev. Mol. Cell Biol. 6, 209–220 10.1038/nrm1591 15738987

[6] Pomorski T.G. and Menon A.K. (2016) Lipid somersaults: Uncovering the mechanisms of protein-mediated lipid flipping. Prog. Lipid Res. 64, 69–84 10.1016/j.plipres.2016.08.003 27528189 PMC5127727

[7] Watanabe R., Sakuragi T., Noji H. and Nagata S. (2018) Single-molecule analysis of phospholipid scrambling by TMEM16F. Proc. Natl. Acad. Sci. U. S. A. 115, 3066–3071 10.1073/pnas.1717956115 29507235 PMC5866571

[8] Kalienkova V., Clerico Mosina V. and Paulino C. (2021) The Groovy TMEM16 Family: Molecular Mechanisms of Lipid Scrambling and Ion Conduction. J. Mol. Biol. 433, 166941 10.1016/j.jmb.2021.166941 33741412

[9] Pifferi S. and Boccaccio A. (2022) Ca2+-Activated Chloride Channels and Phospholipid Scramblases. Int. J. Mol. Sci. 23, 10.3390/ijms23042158 35216275 PMC8875746

[10] Khelashvili G. and Menon A.K. (2022) Phospholipid Scrambling by G Protein-Coupled Receptors. Annu. Rev. Biophys. 51, 39–61 10.1146/annurev-biophys-090821-083030 34932914 PMC9521775

[11] Zwaal R.F., Comfurius P. and van Deenen L.L. (1977) Membrane asymmetry and blood coagulation. Nature 268, 358–360 10.1038/268358a0 887167

[12] Fadok V.A., Voelker D.R., Campbell P.A., Cohen J.J., Bratton D.L. and Henson P.M. (1992) Exposure of phosphatidylserine on the surface of apoptotic lymphocytes triggers specific recognition and removal by macrophages. J. Immunol. 148, 2207–2216 10.4049/jimmunol.148.7.2207 1545126

[13] Chua B.A., Ngo J.A., Situ K. and Morizono K. (2019) Roles of phosphatidylserine exposed on the viral envelope and cell membrane in HIV-1 replication. Cell Commun. Signal 17, 132 10.1186/s12964-019-0452-1 31638994 PMC6805584

[14] López-Marqués R.L., Gourdon P., Günther Pomorski T. and Palmgren M. (2020) The transport mechanism of P4 ATPase lipid flippases. Biochem. J. 477, 3769–3790 10.1042/BCJ20200249 33045059

[15] Lyons J.A., Timcenko M., Dieudonné T., Lenoir G. and Nissen P. (2020) P4-ATPases: how an old dog learnt new tricks - structure and mechanism of lipid flippases. Curr. Opin. Struct. Biol. 63, 65–73 10.1016/j.sbi.2020.04.001 32492637

[16] Ford R.C. and Beis K. (2019) Learning the ABCs one at a time: structure and mechanism of ABC transporters. Biochem. Soc. Trans. 47, 23–36 10.1042/BST20180147 30626703

[17] Thomas C. and Tampé R. (2020) Structural and Mechanistic Principles of ABC Transporters. Annu. Rev. Biochem. 89, 605–636 10.1146/annurev-biochem-011520-105201 32569521

[18] Tadini-Buoninsegni F., Mikkelsen S.A., Mogensen L.S., Holm R., Molday R.S. and Andersen J.P. (2022) Electrogenic reaction step and phospholipid translocation pathway of the mammalian P4-ATPase ATP8A2. FEBS Lett. 597, 495–503 10.1002/1873-3468.14459 35945663

[19] López-Martín M., Renault P., Giraldo J., Vázquez-Ibar J.L. and Perálvarez-Marín A. (2022) In Silico Assessment of the Lipid Fingerprint Signature of ATP2, the Essential P4-ATPase of Malaria Parasites. Membranes (Basel) 12, 702 10.3390/membranes12070702 35877905 PMC9325222

[20] Barreto-Ojeda E., Corradi V., Gu R.-X. and Tieleman D.P. (2018) Coarse-grained molecular dynamics simulations reveal lipid access pathways in P-glycoprotein. J. Gen. Physiol. 150, 417–429 10.1085/jgp.201711907 29437858 PMC5839720

[21] Bechara C., Nöll A., Morgner N., Degiacomi M.T., Tampé R. and Robinson C.V. (2015) A subset of annular lipids is linked to the flippase activity of an ABC transporter. Nat. Chem. 7, 255–262 10.1038/nchem.2172 25698336

[22] Saito K., Fujimura-Kamada K., Furuta N., Kato U., Umeda M. and Tanaka K. (2004) Cdc50p, a protein required for polarized growth, associates with the Drs2p P-type ATPase implicated in phospholipid translocation in Saccharomyces cerevisiae. Mol. Biol. Cell 15, 3418–3432 10.1091/mbc.e03-11-0829 15090616 PMC452594

[23] van der Velden L.M., Wichers C.G.K., van Breevoort A.E.D., Coleman J.A., Molday R.S., Berger R. et al. (2010) Heteromeric interactions required for abundance and subcellular localization of human CDC50 proteins and class 1 P4-ATPases. J. Biol. Chem. 285, 40088–40096 10.1074/jbc.M110.139006 20947505 PMC3000991

[24] Coleman J.A. and Molday R.S. (2011) Critical role of the beta-subunit CDC50A in the stable expression, assembly, subcellular localization, and lipid transport activity of the P4-ATPase ATP8A2. J. Biol. Chem. 286, 17205–17216 10.1074/jbc.M111.229419 21454556 PMC3089563

[25] López-Marqués R.L., Poulsen L.R., Hanisch S., Meffert K., Buch-Pedersen M.J., Jakobsen M.K. et al. (2010) Intracellular Targeting Signals and Lipid Specificity Determinants of the ALA/ALIS P4-ATPase Complex Reside in the Catalytic ALA α-Subunit. Mol. Biol. Cell 21, 791–801 10.1091/mbc.e09-08-0656 20053675 PMC2828965

[26] Víglaš J. and Olejníková P. (2021) An update on ABC transporters of filamentous fungi - from physiological substrates to xenobiotics. Microbiol. Res. 246, 126684 10.1016/j.micres.2020.126684 33529790

[27] Tarling E.J., de Aguiar Vallim T.Q. and Edwards P.A. (2013) Role of ABC transporters in lipid transport and human disease. Trends Endocrinol. Metab. 24, 342–350 10.1016/j.tem.2013.01.006 23415156 PMC3659191

[28] Rizzo J., Stanchev L.D., da Silva V.K.A., Nimrichter L., Pomorski T.G. and Rodrigues M.L. (2019) Role of lipid transporters in fungal physiology and pathogenicity. Comput. Struct. Biotechnol. J. 17, 1278–1289 10.1016/j.csbj.2019.09.001 31921394 PMC6944739

[29] Neumann J., Rose-Sperling D. and Hellmich U.A. (2017) Diverse relations between ABC transporters and lipids: An overview. Biochim. Biophys. Acta Biomembr. 1859, 605–618 10.1016/j.bbamem.2016.09.023 27693344

[30] Pomorski T. and Menon A.K. (2006) Lipid flippases and their biological functions. Cell. Mol. Life Sci. 63, 2908–2921 10.1007/s00018-006-6167-7 17103115 PMC11136118

[31] Nosol K., Bang-Sørensen R., Irobalieva R.N., Erramilli S.K., Stieger B., Kossiakoff A.A. et al. (2021) Structures of ABCB4 provide insight into phosphatidylcholine translocation. Proc. Natl. Acad. Sci. U. S. A. 118, e2106702118 10.1073/pnas.2106702118 34385322 PMC8379956

[32] Park J.G., Kim S., Jang E., Choi S.H., Han H., Ju S. et al. (2022) The lysosomal transporter TAPL has a dual role as peptide translocator and phosphatidylserine floppase. Nat. Commun. 13, 5851 10.1038/s41467-022-33593-2 36195619 PMC9532399

[33] Mi W., Li Y., Yoon S.H., Ernst R.K., Walz T. and Liao M. (2017) Structural basis of MsbA-mediated lipopolysaccharide transport. Nature 549, 233–237 10.1038/nature23649 28869968 PMC5759761

[34] Prescher M., Bonus M., Stindt J., Keitel-Anselmino V., Smits S.H.J., Gohlke H. et al. (2021) Evidence for a credit-card-swipe mechanism in the human PC floppase ABCB4. Structure 29, 1144.e5–1155.e5 10.1016/j.str.2021.05.013 34107287

[35] Perez C., Gerber S., Boilevin J., Bucher M., Darbre T., Aebi M. et al. (2015) Structure and mechanism of an active lipid-linked oligosaccharide flippase. Nature 524, 433–438 10.1038/nature14953 26266984

[36] Alaimo C., Catrein I., Morf L., Marolda C.L., Callewaert N., Valvano M.A. et al. (2006) Two distinct but interchangeable mechanisms for flipping of lipid-linked oligosaccharides. EMBO J. 25, 967–976 10.1038/sj.emboj.7601024 16498400 PMC1409731

[37] Qian H., Zhao X., Cao P., Lei J., Yan N. and Gong X. (2017) Structure of the Human Lipid Exporter ABCA1. Cell 169, 1228.e10–1239.e10 10.1016/j.cell.2017.05.020 28602350

[38] Mangaraj M., Nanda R. and Panda S. (2016) Apolipoprotein A-I: A Molecule of Diverse Function. Indian J. Clin. Biochem. 31, 253–259 10.1007/s12291-015-0513-1 27382195 PMC4910842

[39] Matsuo M. (2022) ABCA1 and ABCG1 as potential therapeutic targets for the prevention of atherosclerosis. J. Pharmacol. Sci. 148, 197–203 10.1016/j.jphs.2021.11.005 35063134

[40] Segrest J.P., Tang C., Song H.D., Jones M.K., Davidson W.S., Aller S.G. et al. (2022) ABCA1 is an extracellular phospholipid translocase. Nat. Commun. 13, 4812 10.1038/s41467-022-32437-3 35974019 PMC9381790

[41] Seigneuret M. and Devaux P.F. (1984) ATP-dependent asymmetric distribution of spin-labeled phospholipids in the erythrocyte membrane: relation to shape changes. Proc. Natl. Acad. Sci. U. S. A. 81, 3751–3755 10.1073/pnas.81.12.3751 6587389 PMC345297

[42] Kean L.S., Fuller R.S. and Nichols J.W. (1993) Retrograde lipid traffic in yeast: identification of two distinct pathways for internalization of fluorescent-labeled phosphatidylcholine from the plasma membrane. J. Cell Biol. 123, 1403–1419 10.1083/jcb.123.6.1403 8253840 PMC2290883

[43] Pagano R.E. and Sleight R.G. (1985) Defining lipid transport pathways in animal cells. Science 229, 1051–1057 10.1126/science.4035344 4035344

[44] Pomorski T., Muller P., Zimmermann B., Burger K., Devaux P.F. and Herrmann A. (1996) Transbilayer movement of fluorescent and spin-labeled phospholipids in the plasma membrane of human fibroblasts: a quantitative approach. J. Cell Sci. 109, 687–698 10.1242/jcs.109.3.687 8907713

[45] Xu P., Okkeri J., Hanisch S., Hu R.-Y., Xu Q., Pomorski T.G. et al. (2009) Identification of a novel mouse P4-ATPase family member highly expressed during spermatogenesis. J. Cell Sci. 122, 2866–2876 10.1242/jcs.047423 19657017

[46] Poulsen L.R., López-Marqués R.L., Pedas P.R., McDowell S.C., Brown E., Kunze R. et al. (2015) A phospholipid uptake system in the model plant Arabidopsis thaliana. Nat. Commun. 6, 7649 10.1038/ncomms8649 26212235

[47] Stanchev L.D., Rizzo J., Peschel R., Pazurek L.A., Bredegaard L., Veit S. et al. (2021) P-Type ATPase Apt1 of the Fungal Pathogen Cryptococcus neoformans Is a Lipid Flippase of Broad Substrate Specificity. J. Fungi (Basel) 7, 10.3390/jof7100843 34682264 PMC8537059

[48] Veit S., Laerbusch S., López-Marqués R.L. and Günther Pomorski T. (2023) Functional Analysis of the P-Type ATPases Apt2-4 from Cryptococcus neoformans by Heterologous Expression in Saccharomyces cerevisiae. J. Fungi (Basel) 9, jof9020202 10.3390/jof9020202 PMC9966271 36836316

[49] Jensen M.S., Costa S.R., Theorin L., Christensen J.P., Pomorski T.G. and López-Marqués R.L. (2016) Application of image cytometry to characterize heterologous lipid flippases in yeast. Cytometry A 89, 673–680 10.1002/cyto.a.22886 27272389

[50] Herrera S.A., Grifell-Junyent M. and Pomorski T.G. (2022) NBD-lipid Uptake Assay for Mammalian Cell Lines. Bio. Protoc. 12, e4330 10.21769/BioProtoc.4330 35340299 PMC8899549

[51] Smit J.J., Schinkel A.H., Oude Elferink R.P., Groen A.K., Wagenaar E., van Deemter L. et al. (1993) Homozygous disruption of the murine mdr2 P-glycoprotein gene leads to a complete absence of phospholipid from bile and to liver disease. Cell 75, 451–462 10.1016/0092-8674(93)90380-9 8106172

[52] van Helvoort A., Smith A.J., Sprong H., Fritzsche I., Schinkel A.H., Borst P. et al. (1996) MDR1 P-glycoprotein is a lipid translocase of broad specificity, while MDR3 P-glycoprotein specifically translocates phosphatidylcholine. Cell 87, 507–517 10.1016/S0092-8674(00)81370-7 8898203

[53] Pohl A., Lage H., Müller P., Pomorski T. and Herrmann A. (2002) Transport of phosphatidylserine via MDR1 (multidrug resistance 1)P-glycoprotein in a human gastric carcinoma cell line. Biochem. J. 365, 259–268 10.1042/bj20011880 12071854 PMC1222671

[54] Shin H.-W. and Takatsu H. (2019) Substrates of P4-ATPases: beyond aminophospholipids (phosphatidylserine and phosphatidylethanolamine). FASEB J. 33, 3087–3096 10.1096/fj.201801873R 30509129

[55] Bosch I., Dunussi-Joannopoulos K., Wu R.L., Furlong S.T. and Croop J. (1997) Phosphatidylcholine and phosphatidylethanolamine behave as substrates of the human MDR1 P-glycoprotein. Biochemistry 36, 5685–5694 10.1021/bi962728r 9153408

[56] Raggers R.J., Vogels I. and van Meer G. (2001) Multidrug-resistance P-glycoprotein (MDR1) secretes platelet-activating factor. Biochem. J. 357, 859–865 10.1042/bj3570859 11463358 PMC1222017

[57] Smith J.D., Le Goff W., Settle M., Brubaker G., Waelde C., Horwitz A. et al. (2004) ABCA1 mediates concurrent cholesterol and phospholipid efflux to apolipoprotein A-I. J. Lipid Res. 45, 635–644 10.1194/jlr.M300336-JLR200 14703508

[58] Hozoji M., Kimura Y., Kioka N. and Ueda K. (2009) Formation of two intramolecular disulfide bonds is necessary for ApoA-I-dependent cholesterol efflux mediated by ABCA1. J. Biol. Chem. 284, 11293–11300 10.1074/jbc.M900580200 19258317 PMC2670134

[59] Vaughan A.M., Tang C. and Oram J.F. (2009) ABCA1 mutants reveal an interdependency between lipid export function, apoA-I binding activity, and Janus kinase 2 activation. J. Lipid Res. 50, 285–292 10.1194/jlr.M800366-JLR200 18776170 PMC2636916

[60] Gulshan K., Brubaker G., Conger H., Wang S., Zhang R., Hazen S.L. et al. (2016) PI(4,5)P2 Is Translocated by ABCA1 to the Cell Surface Where It Mediates Apolipoprotein A1 Binding and Nascent HDL Assembly. Circ. Res. 119, 827–838 10.1161/CIRCRESAHA.116.308856 27514935 PMC5026623

[61] Ujhazy P., Ortiz D., Misra S., Li S., Moseley J., Jones H. et al. (2001) Familial intrahepatic cholestasis 1: studies of localization and function. Hepatology 34, 768–775 10.1053/jhep.2001.27663 11584374

[62] Paulusma C.C., Folmer D.E., Ho-Mok K.S., de Waart D.R., Hilarius P.M., Verhoeven A.J. et al. (2008) ATP8B1 requires an accessory protein for endoplasmic reticulum exit and plasma membrane lipid flippase activity. Hepatology 47, 268–278 10.1002/hep.21950 17948906

[63] Muñoz-Martínez F., Torres C., Castanys S. and Gamarro F. (2010) CDC50A plays a key role in the uptake of the anticancer drug perifosine in human carcinoma cells. Biochem. Pharmacol. 80, 793–800 10.1016/j.bcp.2010.05.017 20510206

[64] Ray N.B., Durairaj L., Chen B.B., McVerry B.J., Ryan A.J., Donahoe M. et al. (2010) Dynamic regulation of cardiolipin by the lipid pump Atp8b1 determines the severity of lung injury in experimental pneumonia. Nat. Med. 16, 1120–1127 10.1038/nm.2213 20852622 PMC4500192

[65] Paulusma C.C., Houwen R.H.J. and Williamson P.L. (2011) The flip side of cardiolipin import. Nat. Med. 17, 413, Author Reply 413-4 10.1038/nm0411-413a 21475228

[66] Takatsu H., Tanaka G., Segawa K., Suzuki J., Nagata S., Nakayama K. et al. (2014) Phospholipid flippase activities and substrate specificities of human type IV P-type ATPases localized to the plasma membrane. J. Biol. Chem. 289, 33543–33556 10.1074/jbc.M114.593012 25315773 PMC4246107

[67] Segawa K., Kurata S. and Nagata S. (2016) Human Type IV P-type ATPases That Work as Plasma Membrane Phospholipid Flippases and Their Regulation by Caspase and Calcium. J. Biol. Chem. 291, 762–772 10.1074/jbc.M115.690727 26567335 PMC4705396

[68] Choy B.C., Cater R.J., Mancia F. and Pryor E.E. (2021) A 10-year meta-analysis of membrane protein structural biology: Detergents, membrane mimetics, and structure determination techniques. Biochim. Biophys. Acta Biomembr. 1863, 183533 10.1016/j.bbamem.2020.183533 33340490 PMC7856071

[69] Pick H., Alves A.C. and Vogel H. (2018) Single-Vesicle Assays Using Liposomes and Cell-Derived Vesicles: From Modeling Complex Membrane Processes to Synthetic Biology and Biomedical Applications. Chem. Rev. 118, 8598–8654 10.1021/acs.chemrev.7b00777 30153012

[70] Amati A.M., Graf S., Deutschmann S., Dolder N. and von Ballmoos C. (2020) Current problems and future avenues in proteoliposome research. Biochem. Soc. Trans. 48, 1473–1492 10.1042/BST20190966 32830854

[71] Rigaud J.L., Pitard B. and Levy D. (1995) Reconstitution of membrane proteins into liposomes: application to energy-transducing membrane proteins. Biochim. Biophys. Acta 1231, 223–246 10.1016/0005-2728(95)00091-V 7578213

[72] Rigaud J.-L., Levy D., Mosser G. and Lambert O. (1998) Detergent removal by non-polar polystyrene beads. Eur. Biophys. J. 27, 305–319 10.1007/s002490050138

[73] Rigaud J.-L. and Lévy D. (2003) Reconstitution of membrane proteins into liposomes. Meth Enzymol. 372, 65–86 10.1016/S0076-6879(03)72004-7 14610807

[74] Zhou X., Sebastian T.T. and Graham T.R. (2013) Auto-inhibition of Drs2p, a yeast phospholipid flippase, by its carboxyl-terminal tail. J. Biol. Chem. 288, 31807–31815 10.1074/jbc.M113.481986 24045945 PMC3814774

[75] Huang Z., Chang X., Riordan J.R. and Huang Y. (2004) Fluorescent modified phosphatidylcholine floppase activity of reconstituted multidrug resistance-associated protein MRP1. Biochim. Biophys. Acta 1660, 155–163 10.1016/j.bbamem.2003.11.010 14757231

[76] Guo D., Singh H., Shimoyama A., Guffick C., Tang Y., Rowe S.M. et al. (2021) Energetics of lipid transport by the ABC transporter MsbA is lipid dependent. Commun. Biol. 4, 1379 10.1038/s42003-021-02902-8 34887543 PMC8660845

[77] Tadini-Buoninsegni F., Mikkelsen S.A., Mogensen L.S., Molday R.S. and Andersen J.P. (2019) Phosphatidylserine flipping by the P4-ATPase ATP8A2 is electrogenic. Proc. Natl. Acad. Sci. U. S. A. 116, 16332–16337 10.1073/pnas.1910211116 31371510 PMC6697784

[78] Romsicki Y. and Sharom F.J. (2001) Phospholipid flippase activity of the reconstituted P-glycoprotein multidrug transporter. Biochemistry 40, 6937–6947 10.1021/bi0024456 11389609

[79] Paweletz L.C., Veit S. and Pomorski T.G. (2022) A Fluorescence-based Approach Utilizing Self-labeling Enzyme Tags to Determine Protein Orientation in Large Unilamellar Vesicles. Bio Protoc. 12, 10.21769/BioProtoc.4542 36505029 PMC9711944

[80] Veit S., Paweletz L.C. and Günther Pomorski T. (2023) Determination of membrane protein orientation upon liposomal reconstitution down to the single vesicle level. Biol. Chem. 10.1515/hsz-2022-0325 36857289

[81] Gutiérrez-Merino C., Bonini de Romanelli I.C., Pietrasanta L.I. and Barrantes F.J. (1995) Preferential distribution of the fluorescent phospholipid probes NBD-phosphatidylcholine and rhodamine-phosphatidylethanolamine in the exofacial leaflet of acetylcholine receptor-rich membranes from Torpedo marmorata. Biochemistry 34, 4846–4855 10.1021/bi00014a042 7718591

[82] Quazi F. and Molday R.S. (2013) Differential phospholipid substrates and directional transport by ATP-binding cassette proteins ABCA1, ABCA7, and ABCA4 and disease-causing mutants. J. Biol. Chem. 288, 34414–34426 10.1074/jbc.M113.508812 24097981 PMC3843056

[83] Margolles A., Putman M., van Veen H.W. and Konings W.N. (1999) The purified and functionally reconstituted multidrug transporter LmrA of Lactococcus lactis mediates the transbilayer movement of specific fluorescent phospholipids. Biochemistry 38, 16298–16306 10.1021/bi990855s 10587454

[84] Wang J., Sun F., Zhang D.-W., Ma Y., Xu F., Belani J.D. et al. (2006) Sterol transfer by ABCG5 and ABCG8: in vitro assay and reconstitution. J. Biol. Chem. 281, 27894–27904 10.1074/jbc.M605603200 16867993 PMC4527585

[85] Wang J., Zhang D.-W., Lei Y., Xu F., Cohen J.C., Hobbs H.H. et al. (2008) Purification and reconstitution of sterol transfer by native mouse ABCG5 and ABCG8. Biochemistry 47, 5194–5204 10.1021/bi800292v 18402465 PMC2707165

[86] Coleman J.A., Kwok M.C.M. and Molday R.S. (2009) Localization, purification, and functional reconstitution of the P4-ATPase Atp8a2, a phosphatidylserine flippase in photoreceptor disc membranes. J. Biol. Chem. 284, 32670–32679 10.1074/jbc.M109.047415 19778899 PMC2781682

[87] Zhou X. and Graham T.R. (2009) Reconstitution of phospholipid translocase activity with purified Drs2p, a type-IV P-type ATPase from budding yeast. Proc. Natl. Acad. Sci. U. S. A. 106, 16586–16591 10.1073/pnas.0904293106 19805341 PMC2757829

[88] Wang J., Molday L.L., Hii T., Coleman J.A., Wen T., Andersen J.P. et al. (2018) Proteomic Analysis and Functional Characterization of P4-ATPase Phospholipid Flippases from Murine Tissues. Sci. Rep. 8, 10795 10.1038/s41598-018-29108-z 30018401 PMC6050252

[89] Ding J., Wu Z., Crider B.P., Ma Y., Li X., Slaughter C. et al. (2000) Identification and functional expression of four isoforms of ATPase II, the putative aminophospholipid translocase. Effect of isoform variation on the ATPase activity and phospholipid specificity. J. Biol. Chem. 275, 23378–23386 10.1074/jbc.M910319199 10801890

[90] Paterson J.K., Renkema K., Burden L., Halleck M.S., Schlegel R.A., Williamson P. et al. (2006) Lipid specific activation of the murine P4-ATPase Atp8a1 (ATPase II). Biochemistry 45, 5367–5376 10.1021/bi052359b 16618126

[91] Theorin L., Faxén K., Sørensen D.M., Migotti R., Dittmar G., Schiller J. et al. (2019) The lipid head group is the key element for substrate recognition by the P4 ATPase ALA2: a phosphatidylserine flippase. Biochem. J. 476, 783–794 10.1042/BCJ20180891 30755463 PMC6402034

[92] Azouaoui H., Montigny C., Dieudonné T., Champeil P., Jacquot A., Vázquez-Ibar J.L. et al. (2017) High phosphatidylinositol 4-phosphate (PI4P)-dependent ATPase activity for the Drs2p-Cdc50p flippase after removal of its N- and C-terminal extensions. J. Biol. Chem. 292, 7954–7970 10.1074/jbc.M116.751487 28302728 PMC5427273

[93] Bai L., Kovach A., You Q., Hsu H.-C., Zhao G. and Li H. (2019) Autoinhibition and activation mechanisms of the eukaryotic lipid flippase Drs2p-Cdc50p. Nat. Commun. 10, 4142 10.1038/s41467-019-12191-9 31515475 PMC6742660

[94] Timcenko M., Lyons J.A., Januliene D., Ulstrup J.J., Dieudonné T., Montigny C. et al. (2019) Structure and autoregulation of a P4-ATPase lipid [Structure and autoregulation of a P4-ATPase lipid flippase]. Nature 571, 366–370 10.1038/s41586-019-1344-7 31243363

[95] Dieudonné T., Herrera S.A., Laursen M.J., Lejeune M., Stock C., Slimani K. et al. (2022) Autoinhibition and regulation by phosphoinositides of ATP8B1, a human lipid flippase associated with intrahepatic cholestatic disorders. Elife 11, e75272 10.7554/eLife.75272 35416773 PMC9045818

[96] Lamy A., Macarini-Bruzaferro E., Dieudonné T., Perálvarez-Marín A., Lenoir G., Montigny C. et al. (2021) ATP2, The essential P4-ATPase of malaria parasites, catalyzes lipid-stimulated ATP hydrolysis in complex with a Cdc50 β-subunit. Emerg. Microbes Infect. 10, 132–147 10.1080/22221751.2020.1870413 33372863 PMC7832587

[97] Nakano K., Yamamoto T., Kishimoto T., Noji T. and Tanaka K. (2008) Protein kinases Fpk1p and Fpk2p are novel regulators of phospholipid asymmetry. Mol. Biol. Cell 19, 1783–1797 10.1091/mbc.e07-07-0646 18199685 PMC2291408

[98] Roelants F.M., Baltz A.G., Trott A.E., Fereres S. and Thorner J. (2010) A protein kinase network regulates the function of aminophospholipid flippases. Proc. Natl. Acad. Sci. U. S. A. 107, 34–39 10.1073/pnas.0912497106 19966303 PMC2806694

[99] Frøsig M.M., Costa S.R., Liesche J., Østerberg J.T., Hanisch S., Nintemann S. et al. (2020) Pseudohyphal growth in Saccharomyces cerevisiae involves protein kinase-regulated lipid flippases. J. Cell Sci. 133, 10.1242/jcs.235994 32661085

[100] Tsai P.-C., Hsu J.-W., Liu Y.-W., Chen K.-Y. and Lee F.-J.S. (2013) Arl1p regulates spatial membrane organization at the trans-Golgi network through interaction with Arf-GEF Gea2p and flippase Drs2p. Proc. Natl. Acad. Sci. U. S. A. 110, E668–E677 10.1073/pnas.1221484110 23345439 PMC3581910

[101] Eckford P.D.W. and Sharom F.J. (2005) The reconstituted P-glycoprotein multidrug transporter is a flippase for glucosylceramide and other simple glycosphingolipids. Biochem. J. 389, 517–526 10.1042/BJ20050047 15799713 PMC1175130

[102] Quazi F., Lenevich S. and Molday R.S. (2012) ABCA4 is an N-retinylidene-phosphatidylethanolamine and phosphatidylethanolamine importer. Nat. Commun. 3, 925 10.1038/ncomms1927 22735453 PMC3871175

[103] Marek M., Milles S., Schreiber G., Daleke D.L., Dittmar G., Herrmann A. et al. (2011) The yeast plasma membrane ATP binding cassette (ABC) transporter Aus1: purification, characterization, and the effect of lipids on its activity. J. Biol. Chem. 286, 21835–21843 10.1074/jbc.M111.244525 21521689 PMC3122238

[104] Traïkia M., Warschawski D.E., Lambert O., Rigaud J.-L. and Devaux P.F. (2002) Asymmetrical membranes and surface tension. Biophys. J. 83, 1443–1454 10.1016/S0006-3495(02)73915-5 12202370 PMC1302243

[105] Shukla S. and Baumgart T. (2021) Enzymatic trans-bilayer lipid transport: Mechanisms, efficiencies, slippage, and membrane curvature. Biochim. Biophys. Acta Biomembr. 1863, 183534 10.1016/j.bbamem.2020.183534 33340491 PMC8351443

[106] Goren M.A., Morizumi T., Menon I., Joseph J.S., Dittman J.S., Cherezov V. et al. (2014) Constitutive phospholipid scramblase activity of a G protein-coupled receptor. Nat. Commun. 5, 5115 10.1038/ncomms6115 25296113 PMC4198942

[107] Dietel L., Kalie L. and Heerklotz H. (2020) Lipid Scrambling Induced by Membrane-Active Substances. Biophys. J. 119, 767–779 10.1016/j.bpj.2020.07.004 32738218 PMC7451870

[108] Papadopulos A., Vehring S., López-Montero I., Kutschenko L., Stöckl M., Devaux P.F. et al. (2007) Flippase activity detected with unlabeled lipids by shape changes of giant unilamellar vesicles. J. Biol. Chem. 282, 15559–15568 10.1074/jbc.M604740200 17369612

[109] Wang L., Iwasaki Y., Andra K.K., Pandey K., Menon A.K. and Bütikofer P. (2018) Scrambling of natural and fluorescently tagged phosphatidylinositol by reconstituted G protein-coupled receptor and TMEM16 scramblases. J. Biol. Chem. 293, 18318–18327 10.1074/jbc.RA118.004213 30287690 PMC6254352

[110] Matoba K., Kotani T., Tsutsumi A., Tsuji T., Mori T., Noshiro D. et al. (2020) Atg9 is a lipid scramblase that mediates autophagosomal membrane expansion. Nat. Struct. Mol. Biol. 27, 1185–1193 10.1038/s41594-020-00518-w 33106658

[111] Ezanno P., Cribier S. and Devaux P.F. (2010) Asymmetrical stress generated by the erythrocyte lipid flippase triggers multiple bud formation on the surface of spherical giant liposomes. Eur. Biophys. J. 39, 1277–1280 10.1007/s00249-009-0557-3 19937014

[112] Mathiassen P.P.M., Menon A.K. and Pomorski T.G. (2021) Endoplasmic reticulum phospholipid scramblase activity revealed after protein reconstitution into giant unilamellar vesicles containing a photostable lipid reporter. Sci. Rep. 11, 14364 10.1038/s41598-021-93664-0 34257324 PMC8277826

[113] López-Montero I., Rodriguez N., Cribier S., Pohl A., Vélez M. and Devaux P.F. (2005) Rapid transbilayer movement of ceramides in phospholipid vesicles and in human erythrocytes. J. Biol. Chem. 280, 25811–25819 10.1074/jbc.M412052200 15883154

[114] Marquardt D., Heberle F.A., Miti T., Eicher B., London E., Katsaras J. et al. (2017) 1H NMR Shows Slow Phospholipid Flip-Flop in Gel and Fluid Bilayers. Langmuir 33, 3731–3741 10.1021/acs.langmuir.6b04485 28106399 PMC5397887

[115] Nakano M., Fukuda M., Kudo T., Endo H. and Handa T. (2007) Determination of interbilayer and transbilayer lipid transfers by time-resolved small-angle neutron scattering. Phys. Rev. Lett. 98, 238101 10.1103/PhysRevLett.98.238101 17677937

[116] Steinkühler J., de Tillieux P., Knorr R.L., Lipowsky R. and Dimova R. (2018) Charged giant unilamellar vesicles prepared by electroformation exhibit nanotubes and transbilayer lipid asymmetry. Sci. Rep. 8, 11838 10.1038/s41598-018-30286-z 30087440 PMC6081385

[117] Carvalho K., Ramos L., Roy C. and Picart C. (2008) Giant unilamellar vesicles containing phosphatidylinositol(4,5)bisphosphate: characterization and functionality. Biophys. J. 95, 4348–4360 10.1529/biophysj.107.126912 18502807 PMC2567945

[118] Markones M., Drechsler C., Kaiser M., Kalie L., Heerklotz H. and Fiedler S. (2018) Engineering Asymmetric Lipid Vesicles: Accurate and Convenient Control of the Outer Leaflet Lipid Composition. Langmuir 34, 1999–2005 10.1021/acs.langmuir.7b03189 29294294

[119] Markones M., Fippel A., Kaiser M., Drechsler C., Hunte C. and Heerklotz H. (2020) Stairway to Asymmetry: Five Steps to Lipid-Asymmetric Proteoliposomes. Biophys. J. 118, 294–302 10.1016/j.bpj.2019.10.043 31843262 PMC6976795

[120] Veit S., Paweletz L.C., Bohr S.S.-R., Menon A.K., Hatzakis N.S. and Pomorski T.G. (2022) Single Vesicle Fluorescence-Bleaching Assay for Multi-Parameter Analysis of Proteoliposomes by Total Internal Reflection Fluorescence Microscopy. ACS Appl. Mater. Interfaces 14, 29659–29667 10.1021/acsami.2c07454 35748880 PMC11194769

[121] Veshaguri S., Christensen S.M., Kemmer G.C., Ghale G., Møller M.P., Lohr C. et al. (2016) Direct observation of proton pumping by a eukaryotic P-type ATPase. Science 351, 1469–1473 10.1126/science.aad6429 27013734 PMC5023152

[122] Kosmidis E., Shuttle C.G., Preobraschenski J., Ganzella M., Johnson P.J., Veshaguri S. et al. (2022) Regulation of the mammalian-brain V-ATPase through ultraslow mode-switching. Nature 611, 827–834 10.1038/s41586-022-05472-9 36418452 PMC11212661

[123] Chalat M., Moleschi K. and Molday R.S. (2017) C-terminus of the P4-ATPase ATP8A2 functions in protein folding and regulation of phospholipid flippase activity. Mol. Biol. Cell 28, 452–462 10.1091/mbc.e16-06-0453 27932490 PMC5341728

[124] Lee S., Uchida Y., Wang J., Matsudaira T., Nakagawa T., Kishimoto T. et al. (2015) Transport through recycling endosomes requires EHD1 recruitment by a phosphatidylserine translocase. EMBO J. 34, 669–688 10.15252/embj.201489703 25595798 PMC4365035

[125] Auland M.E., Roufogalis B.D., Devaux P.F. and Zachowski A. (1994) Reconstitution of ATP-dependent aminophospholipid translocation in proteoliposomes. Proc. Natl. Acad. Sci. U. S. A. 91, 10938–10942 10.1073/pnas.91.23.10938 7971987 PMC45141

[126] Cheng M.-T., Chen Y., Chen Z.-P., Liu X., Zhang Z., Chen Y. et al. (2022) Structural insights into the activation of autoinhibited human lipid flippase ATP8B1 upon substrate binding. Proc. Natl. Acad. Sci. U. S. A. 119, e2118656119 10.1073/pnas.2118656119 35349344 PMC9168909

[127] Shukla S., Rai V., Saini P., Banerjee D., Menon A.K. and Prasad R. (2007) Candida drug resistance protein 1, a major multidrug ATP binding cassette transporter of Candida albicans, translocates fluorescent phospholipids in a reconstituted system. Biochemistry 46, 12081–12090 10.1021/bi700453e 17924650 PMC2386675

[128] Eckford P.D.W. and Sharom F.J. (2010) The reconstituted Escherichia coli MsbA protein displays lipid flippase activity. Biochem. J. 429, 195–203 10.1042/BJ20100144 20412049 PMC2888566

[129] Arashiki N., Takakuwa Y., Mohandas N., Hale J., Yoshida K., Ogura H. et al. (2016) ATP11C is a major flippase in human erythrocytes and its defect causes congenital hemolytic anemia. Haematologica 101, 559–565 10.3324/haematol.2016.142273 26944472 PMC5004368

